# Prognostic relevance of arterial ammonia levels in different acute and acute-on-chronic liver diseases

**DOI:** 10.1186/cc10997

**Published:** 2012-03-20

**Authors:** V Fuhrmann, A Drolz, B Jaeger, M Wewalka, R Saxa, T Horvatits, T Perkmann, C Zauner, P Ferenci

**Affiliations:** 1 Medical University Vienna, Austria

## Introduction

Increased levels of arterial ammonia in patients with acute liver failure (ALF) are associated with increased mortality. There is a lack of data for prognostic impact of arterial ammonia in patients with acute-on-chronic liver failure (AoCLF) and hypoxic hepatitis (HH). We evaluated arterial ammonia levels and their prognostic relevance in patients with HH, ALF, AoCLF and without evidence for any liver disease.

## Methods

One-hundred and ninety-seven critically ill patients were studied at the Medical University Vienna: 72 patients with HH, 22 with ALF, 58 with AoCLF and 45 critically patients without evidence for liver disease. Arterial ammonia concentrations were assessed on a daily basis in all patients and compared among the four study groups as well as between 28-day survivors and nonsurvivors.

## Results

The 28-day mortality rates in HH, ALF, AoCLF and in the control group were 54% (*n *= 39), 27% (*n *= 6), 53% (*n *= 31) and 27% (*n *= 12), respectively. Peak arterial ammonia levels in patients with HH were significantly higher in 28-day nonsurvivors compared to survivors (*P *< 0.01). Cox regression identified peak arterial ammonia concentrations as an independent predictor for 28-day mortality (*P *< 0.01). Peak arterial ammonia levels in 28-day transplant-free ALF survivors were significantly lower compared to ALF patients who died or underwent liver transplantation (*P *< 0.05). There was no association between outcome and arterial ammonia in AoCLF patients and in the control group.

## Conclusion

Elevated arterial ammonia levels are frequently observed in critically ill patients with liver injury but not in patients of comparable severity of illness without hepatic impairment. They indicate poor prognosis in HH and ALF, but not AoCLF.

